# Adaptation of DNA to Protein Binding Revealed by Spectroscopy and Molecular Simulation

**DOI:** 10.1021/acs.jpcb.5c00189

**Published:** 2025-05-28

**Authors:** Thor van Heesch, Sudhanshu Sharma, Bert van Erp, Alberto Pérez de Alba Ortíz, Remus T. Dame, Jocelyne Vreede, Krishna Gavvala

**Affiliations:** † Van ’t Hoff Institute of Molecular Sciences, 1234 University of Amsterdam, Science Park 904, Amsterdam 1098 XH, The Netherlands; ‡ Department of Chemistry, 233600 Indian Institute of Technology Hyderabad, Kandi, Sangareddy, Telangana 502284, India; § 4496 Leiden Institute of Chemistry, Einsteinweg 55, Leiden 2333 CC, The Netherlands; ∥ Centre for Microbial Cell Biology, Leiden University, Einsteinweg 55, Leiden 2333 CC, The Netherlands; ⊥ Centre for Interdisciplinary Genome Research, Leiden University, Einsteinweg 55, Leiden 2333 CC, The Netherlands; # Informatics Institute, University of Amsterdam, Science Park 904, Amsterdam 1098 XH, The Netherlands; ∇ Centre for Microbial Cell Biology, Leiden University, Einsteinweg 55,, Leiden 2333 CC, The Netherlands

## Abstract

DNA demonstrates remarkable structural diversity, transitioning between conformations such as B-DNA and A-DNA under specific environmental or protein-binding conditions. These transitions are relevant for mediating cellular processes such as gene regulation, DNA organization, and stress response. In bacteria, the histone-like nucleoid structuring protein (H-NS) exemplifies the interaction between sequence-dependent DNA conformational adaptability and protein-mediated regulatory mechanisms. Despite evidence for the strong affinity of H-NS for AT-rich DNA, the specific molecular and structural interactions driving this recognition remain largely unclear. Combining fluorescence spectroscopy, circular dichroism (CD), molecular dynamics (MD) simulations, and enhanced sampling techniques, we show that H-NS exhibits a 10-fold higher affinity for ApT repeats compared to that of GpC repeats. Interestingly, selective binding of H-NS to AT-rich DNA causes a structural adaptation in the DNA, including increased bending flexibility, minor groove widening, and localized A-like DNA features, while GC-rich DNA remains closer to the canonical B-form. Our approach yielded detailed insights into how H-NS exploits the intrinsic conformational plasticity of DNA to achieve sequence-dependent binding. More broadly, this work illustrates how DNA-binding proteins can harness the structural adaptability of the DNA double helix, which may modulate regulatory outcomes, and provides insight into how the intrinsic properties of DNA shape protein–DNA interactions in diverse biological systems.

## Graphical abstract

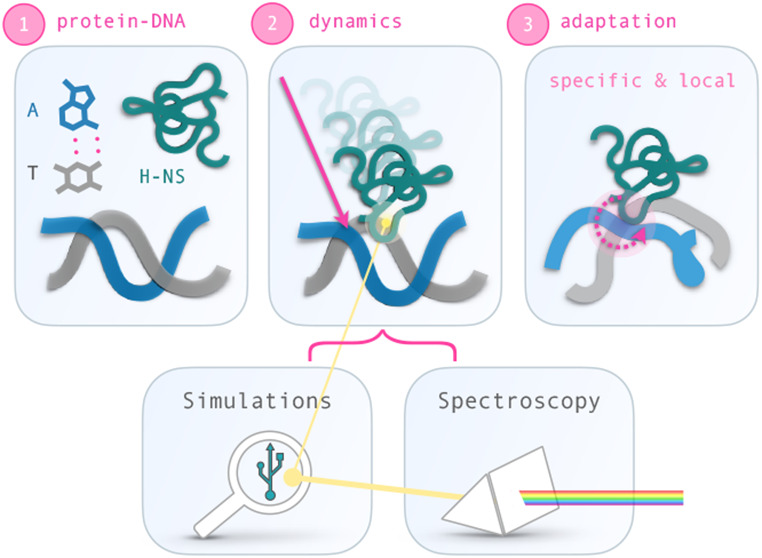

## Introduction

DNA can adopt multiple conformations, with B-DNA and A-DNA being the most prominent right-handed double helical forms. B-DNA is the dominant conformation under physiological conditions, stabilized by the hydrated environment commonly found in cells.^1^ In contrast, A-DNA or some gradual deformation of B-DNA to A-like geometry becomes more prominent under dehydrated conditions, or in specific protein–DNA complexes.^2–4^ Structurally, A-DNA differs from B-DNA by its greater inclination of base pairs relative to the helical axis. Furthermore, A-DNA tends to have a wider minor and narrower major grooves than B-DNA,^5^ and a shift in sugar puckering from the C2’-endo (South) in B-DNA to the C3′-endo (North) accompanied by a negative glycosidic angle shift,^6,7^ see Supplementary Figure S1B. Transitions between these conformations are biologically significant, particularly in protein–DNA interactions, which regulate vital cellular functions associated with numerous types of DNA transactions. In particular, DNA can locally assume the A-form when in complex with proteins such as endonucleases or transcription factors,^8,9^ as well as in cocrystal structures of certain DNA polymerases.^10^ Furthermore, A-DNA has been suggested to play a protective role as A-DNA has a increased resistance to UV radiation, observed in spores of Gram-positive bacteria. A-DNA may serve as a defense mechanism against other forms of DNA damage under stress conditions such as dehydration.^11–13^ This protective function appears to be enhanced by the presence of Small Acid-Soluble Proteins (SASPs), which can induce a transition from B- to A-form as a response to environmental stressors.^11^ Additionally, the A/B equilibrium may play a role in DNA/RNA hybridization, as suggested by observations of pseudo-A-form RNA-DNA hybrids in systems such as CRISPR-Cas9.^14^ Characterizing the factors that influence these structural changes in DNA and how they affect sequence-specific recognition is important to understand the molecular basis of protein–DNA interactions.

The histone-like nucleoid structuring protein (H-NS) is a major component of bacterial chromatin that regulates gene expression.^15–17^ The protein consists of an N-terminal multimerization domain (residues 1–83) and a C-terminal DNA binding domain (DBD, residues 93–137) conserved in DNA organizing proteins in bacteria.^18^ The binding of H-NS to DNA is initiated by the interaction of the DBD with the minor groove.^19^ Binding occurs with a strong preference for AT-rich sequences.^20–22^ The initial binding, which likely occurs at these AT-rich sequences, is followed by H-NS multimerization on DNA through the formation of lateral filaments along DNA or bridged DNA segments, allowing H-NS to coat extensive regions of up to 20 kB in vivo.^23–26^ H-NS preferentially binds to curved DNA, especially regions with short A-tracts interrupted by TA steps, suggesting that the nucleotide sequence and DNA shape play a role in the interaction of H-NS with DNA.^21,23,25,27–29^ Although DBD arrangements in DNA within a filament differ for bridged and lateral filaments, the contact sites in DNA remain unchanged.^30^ The DBDs, both in the lateral and bridged conformations, are irregularly spaced,^31^ made possible by the flexible linkers that allow the DBDs to seek out the tightest AT-rich binding locations within reach.^30^

H-NS binds to the minor groove using a conserved QGR motif (Q112, G113, R114), which allows H-NS to preferentially bind to AT-rich sequences with narrower minor grooves and more negative electrostatic potential that facilitate more stable insertion, especially of the R114 residue.^8,30,32^ This QGR motif is essential for DNA binding and functions similarly to the motifs in H-NS-related proteins, such as Ler and Lsr2.^20,33^ In the vicinity, Trp-109, an intrinsic fluorophore within the DBD, presents an opportunity to monitor changes in the local environment after DNA binding.^34^ Fluorescence spectroscopy studies revealed a blue-shifted emission maximum at 327 nm for H-NS compared to tryptophan in aqueous solution (348 nm), indicating restricted mobility and a buried location in the native structure.^34^ Upon DNA binding, the fluorescence intensity of Trp-109 decreases, suggesting that the local environment around the DBD changes without shifting the emission wavelength. Although fluorescence changes in Trp-109 could indicate adjustments in the local environment surrounding the QGR motif, it is unclear whether these changes correspond to structural adaptations in the DBD, the DNA itself, or both. In particular, it is unknown whether H-NS binding to AT-rich sequences drives localized transitions toward A-like DNA conformations, as has been observed with other protein–DNA interactions.

The molecular mechanisms by which H-NS induces sequence-specific structural changes in DNA, especially in a sequence-specific manner, remain largely unexplored. Although interactions with AT-rich sequences and the minor groove of DNA are implicated, the precise mechanism of H-NS high-affinity site recognition remains unclear.^35^ Despite this, several factors have been identified that influence the binding behavior of H-NS. For example, the type and concentration of cations (e.g., magnesium and potassium) can affect these binding modes; magnesium promotes DNA bridging, while high potassium concentrations disrupt this mode.^36^ H-NS may also prefer binding to undertwisted DNA, i.e., negative supercoiled regions, as suggested by recent genome-scale mapping studies.^37^ This preference aligns with the ability of H-NS to form DNA–DNA bridges that have been shown to constrain negative supercoils^38^ and limit short-range interactions with nearby DNA.^39^

Molecular Dynamics (MD) simulations of multiple H-NS/DNA complexes show that H-NS consistently forms significantly more stable interactions with AT-rich sequences, while GC-rich sequences, having wider minor grooves, present a less favorable electrostatic environment, resulting in weaker and less stable H-NS interactions.^32,35^ Using Steered Molecular Dynamics (SMD) simulations, the largest difference in dissociation free energy between the ApT and GpC steps sequences was found to be 8.5 kcal/mol,^35^ substantiating the more favorable binding of H-NS to AT-rich DNA. To further investigate the sequence-dependent nature of H-NS-DNA interactions, we employed a combination of fluorescence spectroscopy, MD simulations, enhanced sampling techniques, and circular dichroism (CD) spectroscopy. The experimental approaches aimed to quantify binding affinities and identify conformational changes in H-NS DBD and the DNA itself upon binding to an ApT versus a GpC sequence. Fluorescence spectroscopy allowed us to investigate changes in the local environment of Trp-109 after binding to DNA sequences with different base pair compositions. MD simulations in combination with metadynamics^40^ of DNA twist were used to explore the molecular details of these interactions and the structural adaptability of DNA, while CD spectroscopy provided insight into global conformational shifts upon H-NS binding.

Our results reveal a sequence-dependent interaction with the ApT sequences exhibiting higher affinity for H-NS, accompanied by distinctive conformational changes in the DNA characterized by a local transition in the DNA backbone to an A-like conformation. In contrast, the GpC sequence exhibited weaker binding and minimal structural adaptation. These findings are consistent with previous observations that, under subsaturating conditions, H-NS selectively binds to particular DNA regions, with the nucleotide sequence strongly influencing the binding pattern.^41,42^ The findings support the hypothesis that H-NS preferentially binds to AT-rich DNA regions as a result of favorable minor groove characteristics. This selective binding induces localized conformational shifts, which can contribute to the regulation of gene expression by modulating DNA accessibility and structure. Such effects may be related to the mechanisms described for other nucleoid-associated proteins, such as IHF and FIS,^43,44^ which influence DNA topology and propagate torsional stress,^45^ impacting transcription activation and recombination processes. The combination of various spectroscopic techniques and molecular simulation methods enables investigating sequence-dependent protein–DNA interactions, particularly for proteins that recognize or induce structural adaptations in DNA, such as transcription factors, thus informing studies of genome regulation and stability. In the next section, we discuss these results in detail, examining the structural and energetic differences between H-NS interactions with AT-rich versus GC-rich sequences and their implications for understanding the molecular mechanisms.

## Methods

### Sample Preparation

The DNA binding domain (DBD) of H-NS was obtained using a protocol adapted from that described by Shindo et al.^47^ First, H-NS was purified as previously described by van der Valk et al. using pRD18 in strain NT210 derived from Escherichia coli BL21 (laboratory collection) for overexpression.^36^ The full-length H-NS protein was concentrated after purification by loading the protein onto a 1 mL heparin column (GE Healthcare) and using reverse flow with 1 M NaCl in the buffer to elute the protein in a single sharp peak. The peak fraction was loaded onto a pre-equilibrated Superdex G200 increase with trypsin buffer (20 mM Tris-HCl pH 7.2, 300 mM KCl, 10% glycerol) at 4 °C and eluted in fractions. The peak fraction was incubated with trypsin (Sigma-Aldrich, bovine) at 20 °C for 10 min and the resulting protein mixture was loaded onto a pre-equilibrated Superdex G75 increase to separate the H-NS DNA binding domain from trypsin and the N-terminal multimerization domain. The fraction containing the H-NS DNA binding domain was identified using SDS-PAGE. The quality and integrity of the purified protein were verified using intact protein LC-MS. 12-mer 5′-ATATATATATAT-3′ and 5′-GCGCGCGCGCGC-3′ dsDNA sequences were purchased from Integrated DNA Technologies (IDT), India, in lyophilized form. Calf thymus Deoxyribonucleic acid (ct-DNA) was purchased from Merck Life Science Pvt. Ltd. (CAS no. 73049–39–5). 4′,6-diamidino-2-phenylindole (DAPI, CAS no. 28718–90–3) was purchased from Sigma-Aldrich. Tris­(hydroxymethyl)­aminomethane (TRIS) buffer was obtained from Sigma-Aldrich (CAS no. 77–86–1). Hydrochloric acid (HCl, CAS no. 7647–01–0) was obtained from Avra Chemicals Pvt. Ltd. Potassium chloride (KCl, CAS no. 7447–40–7) was obtained from Sisco Research Laboratories Pvt. Ltd.(SRL), India. All measurements were performed in buffer solution with a composition maintained at 20 mM Tris-HCl (pH = 8.0) + 50 mM KCl.

### Steady State Spectroscopy

A JASCO-V730 absorption spectrophotometer was used to calculate the concentration of ct-DNA (ϵ = 6600 M^–1^ cm^–1^ per nucleotide at 260 nm) and DAPI (ϵ = 27,000 M^–1^ cm^–1^ at 353 nm) from their respective molar extinction coefficients. A JASCO FP-8350 spectrofluorometer was used to measure steady-state fluorescence spectra of DAPI and Trp of H-NS. For measuring DAPI emission spectra, the excitation wavelength was set at λ_ex_ = 370 nm, and for measuring excitation spectra, the emission wavelength was set at λ_em_ = 450 nm. In case of Trp emission spectra from H-NS, the excitation wavelength was set at λ_ex_ = 280 nm and for measuring excitation spectra, the emission wavelength was set at λ_em_ = 340 nm. For both of these fluorescence emission and excitation spectra, the scan speed was kept at 200 nm/min, and the slit width was maintained at 10:10 nm. All measurements were performed at 20 mM Tris-HCl pH = 8.0 + 50 mM KCl at 298 K. The fluorescence spectra were corrected from the inner filter effect using the following equation

1 $$F_{\mathrm{corr}}=F_{\mathrm{obs}}\times \exp\left(\left(A_{\mathrm{ex}}+A_{\mathrm{em}}\right)/2\right)$$

where *F*_obs_ is the corrected fluorescence intensity and *F*_obs_ is the observed fluorescence intensity. *A*_ex_ denotes the absorbance value at excitation wavelength and *A*_em_ is the absorbance value at the emission wavelength.^48^ Note that we only utilized UV measurements to correct the inner-filter effect in the fluorescence quenching, as the absorption maxima of the DNA at 260 nm and the protein at 280 nm are too close to reliably extract interaction information.

### Time Resolved Fluorescence Spectroscopy

The fluorescence decay profiles of DAPI were recorded using Time Correlated Single Photon Counting (TCSPC) set up by Horiba (FluoroHub). The instrument response function was 1 ns and the repetition rate was 1 MHz. Horiba’s DAS6 analysis software was utilized to evaluate fluorescence decays and, using multiexponential decay functions, the IRF was convoluted to fit decay profiles using the following equation

2 $$I\left(t\right)=\sum \alpha_{i}e^{-1/\tau_{i}}$$

where *I*(*t*) denotes the time-dependent fluorescence intensity, α_*i*_ are the pre-exponential factors and τ_*i*_ are the fluorescence lifetimes of the *i*th components. All measurements were performed at 20 mM Tris-HCl pH = 8.0 + 50 mM KCl at 298 K.

### Circular Dichroism (CD) Spectroscopy

JASCO-J815 spectrophotometer was utilized to measure the CD spectra of the secondary structure of the DNA sequences. The scan speed was maintained at 200 nm/min for the three types of DNA sequences. The baseline was corrected by a blank solution of 20 mM Tris-HCl pH = 8.0 + 50 mM KCl. The concentration of DNA sequences was maintained at 85 μM per nucleotide.

### Molecular Dynamics Simulations

We used two systems in this study, the DNA binding domain of H-NS (later referred to as H-NS) in complex with two different 12-mer dsDNA nucleotide sequences: 5′-GCATATATATGC-3′ and 5′-GCGCGCGCGCGC-3′, see Supporting Figure S1A for snapshots of the two systems. Note that the ApT sequence is capped with GC base pairs at both ends to reduce the probability of base pair opening at the termini of the DNA strand. We performed Molecular Dynamics (MD) simulations of these systems in explicit water with 50 mM potassium chloride. The initial structure of H-NS was taken from the solution NMR structure of the DNA-binding region of Salmonella typhimurium of H-NS (residues 91–139, PDB code 2L93).^20^ An acetyl cap was placed on the N-terminus to neutralize its charge since this domain is connected to a linker in the full-length protein at the N-terminal end. The coordinates of H-NS bound to the minor groove of the 12-bp high-affinity strand of dsDNA were obtained from earlier work.^19^ To obtain an initial conformation of H-NS bound to the sequences presented in this study, the high-affinity sequence with H-NS bound has been rebuilt with Web 3DNA 2.0 while preserving the geometry of the dsDNA backbone.^49^

The preparation of the system for MD simulations consisted of placing the bare DNA or H-NS bound to DNA in a periodic dodecahedron box, with the box boundaries at least 1.2 nm from the system, resulting in simulation boxes with edges of 7 nm or larger, followed by the addition of water molecules. To mimic experimental conditions^20^ and neutralize the system, we added 50 mM KCl by replacing water molecules with ions. The interactions between atoms are described by the force field AMBER14sb-parmbsc1 ^50,51^ in combination with the TIP3P water model.^52^ We selected this particular force field because it covers the topologies for both amino acids and nucleotides and provides good representations of the static and dynamic properties of DNA under a wide range of conditions.^51^ For nonbonded interactions, both van der Waals and electrostatic, we used a cutoff at 1.1 nm. Long-range electrostatic interactions were handled using the particle mesh Ewald method^53,54^ with a grid spacing of 0.12 nm. To remove unfavorable interactions, we performed energy minimization using steepest descent. By applying position restraints on the heavy atoms of the protein and DNA with a force constant in each direction of 1000 kJ/mol nm^2^ and performing 0.1 ns of MD at a temperature of 298 K and a pressure of 1 bar, we relaxed the water and ions around the initial structures.

After preparation, we performed 3× 1 μs MD runs for DNA bound and unbound with H-NS, varying initial conditions by assigning new random starting velocities drawn from the Maxwell–Boltzmann distribution at 298 K. All simulations were performed with GROMACS, version 2021 ^55,56^ in a locally maintained cluster, with the leapfrog integration scheme and a time step of 2 fs, using LINCS^57^ to constrain protein bonds and SETTLE^58^ to constrain water bonds. All simulations were performed in the isothermal–isobaric ensemble at a pressure of 1 bar, using the v-rescale thermostat^59^ and the isotropic Parrinello–Rahman barostat.^60,61^ For the purpose of analysis, the first 500 ns were omitted. Trajectories are analyzed using MDTraj,^62^ and characterization of A-DNA and calculation of protein–DNA contacts are outlined in the Supporting Information.

### Metadynamics Simulations

Metadynamics production runs are performed with GROMACS 2023.2,^55,56^ patched with PLUMED-2.9.0 ^63,64^ and modified by the added RBB-NA code,^65^ available at: https://github.com/AderikVoorspoels/RBB-NA. The RBB-NA algorithm is capable of controlling rigid base parameters in all-atom simulations of nucleic acids. With suitable bias potentials, this algorithm can “force” a DNA molecule to assume specific values of the six rotational parameters (tilt, roll, twist, buckle, propeller and opening) and/or the six translational parameters (shift, slide, rise, shear, stretch, and stagger). As the collective variable (CV), we used the twist of the central step of the (sixth) base pair. The metadynamics Gaussian potentials were set to have a width of 0.04 radians and a height of 0.1 kJ/mol, and they were deposited every 4 ps. Finally, we applied the bias only within the 10 to 60° interval, setting the bias force equal to zero outside the boundary. We applied this interval to ensure that the DNA did not completely denature, which can happen at extreme twist values. A metadynamics simulation can produce reliable free energy profiles after the system can freely diffuse along the biasing coordinate.^66^ For all systems, we analyzed the twist to verify that the dynamics have reached this free diffusion regime along the bias coordinate, indicating that the free energy profile has been reasonably approximated. This typically occurs before 10 ns of biasing. We obtained metadynamics-based free-energy profiles, i.e., the negative of the sum of Gaussian potentials, every 2 ns. We obtained our final free-energy profiles and standard deviations by averaging the profiles over a window of 40 ns starting at 10 ns of each production run. In some cases, more than one of the central base pairs in the DNA opened (e.g., complete breaking of hydrogen-bond pairs) and did not recover its complementary base pair after opening; in these situations, the data were pruned and excluded from the averaging (see Supporting Figure S2 for time traces of the CV).

## Results and Discussion

### Fluorescence Spectroscopy and Molecular Dynamics Simulations Reveal that H-NS Binds Stronger to AT-Rich DNA

The DNA binding domain (DBD) of the H-NS protein contains an intrinsic fluorophore, tryptophan (Trp), located at position 109 (in older studies often referred to as Trp108). The Trp-109 residue is located within a small loop formed by the QGR motif, which protrudes from the surface of the DBD. This loop structure encases Trp-109, with Gly-111 and Pro-116 as flanking residues. Although arginine (R) and glutamine (Q) are highly hydrophilic, as is the protein backbone, Trp-109 is predominantly hydrophobic due to its large nonpolar indole group. However, it retains a slight amphipathic nature, as the nitrogen atom in the indole side chain can engage in hydrogen bonding. Within the loop formed by the QGR motif and neighboring residues, Trp-109 is largely shielded from the aqueous environment, with its hydrophobic side chain stabilized in this microenvironment. Furthermore, the amino group of Trp-109 exhibits a localized positive partial charge oriented toward the surface of the DBD and, in part, exposed to the solvent. See Figure 1 for a molecular visualization of the H-NS bound to the ApT sequence with the important residues highlighted in the side-panel. Additional snapshots of H-NS in complex with ApT and GpC are provided in the Supporting Information in Supporting Figure S1A.

**Figure 1:**
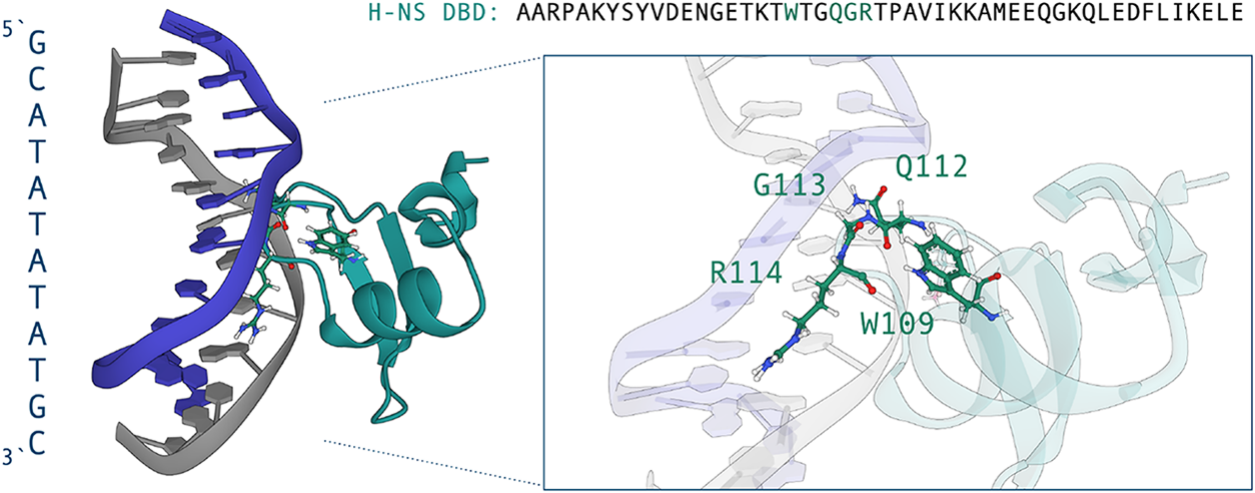
Molecular visualization of H-NS bound to the ApT DNA sequence, based on a snapshot of the molecular dynamics trajectories. The left panel shows the overall structure of the DNA duplex (blue and gray) interacting with the DNA-binding domain (DBD) of H-NS (teal). The right panel provides a zoomed-in view of the key residues involved in the interaction: W109, Q112, G113, and R114. These residues mediate contacts with the DNA bases and backbone, highlighting the specificity of H-NS for ApT-rich sequences. The sequence of the H-NS DBD is shown above for reference, with the region corresponding to the DNA-binding motif highlighted in green. Visualization has been done with Mol*Viewer.^46^ Note that snapshots of H-NS in complex with ApT and GpC are shown in Figure S1A in the Supporting Information.

To study the interaction between H-NS and DNA, we monitored intrinsic fluorescence by exciting Trp-109 at λ_ex_ = 280 nm in the presence of two different DNA sequences: ApT step and the GpC step. The intrinsic fluorescence decay of H-NS is shown in Supporting Figure S4, showing a negligible change in the fluorescence decay upon adding DNA. This observation indicates a static quenching mechanism. As shown in Supporting Figure S5, fluorescence spectra of the H-NS DBD with ApT and GpC sequences reveal that the emission intensity of Trp-109 is quenched when binding to DNA. Similar trends appear in the excitation spectra at the emission maximum (λ_em_ = 340 nm), indicating possible changes in the secondary structure of the protein or direct contact between the DNA strands and Trp-109. These changes may influence the local environment around Trp-109, leading to changes in fluorescence. To characterize the sequence specificity of DBD, we determined the binding constants of the fluorescence quenching data, fitting them to Hill’s equation, see Figure 2. The dissociation constant for the ApT sequence (*K*_D_ = 1.57 ± 0.38 × 10^4^ nM) indicates a higher affinity compared to the GpC sequence (*K*_D_ = 3.35 ± 1.62 × 10^5^ nM). The Hill coefficients for the ApT and GpC sequences, *n*_ApT_ = 1.38 ± 0.10 and *n*_GpC_ = 2.27 ± 0.17, suggest mild cooperativity. The observed difference aligns with previous studies that measured H-NS-DNA interactions using full-length H-NS.^34^ The relative changes in Gibbs free energy, calculated using Δ*G* = −*RT*ln (*K*), are −6.55 kcal/mol for the ApT sequence and −4.74 kcal/mol for the GpC sequence, matching a predicted 40% difference based on predictions of the dissociation free energies, which favors the ApT sequence.^32,35^

**Figure 2:**
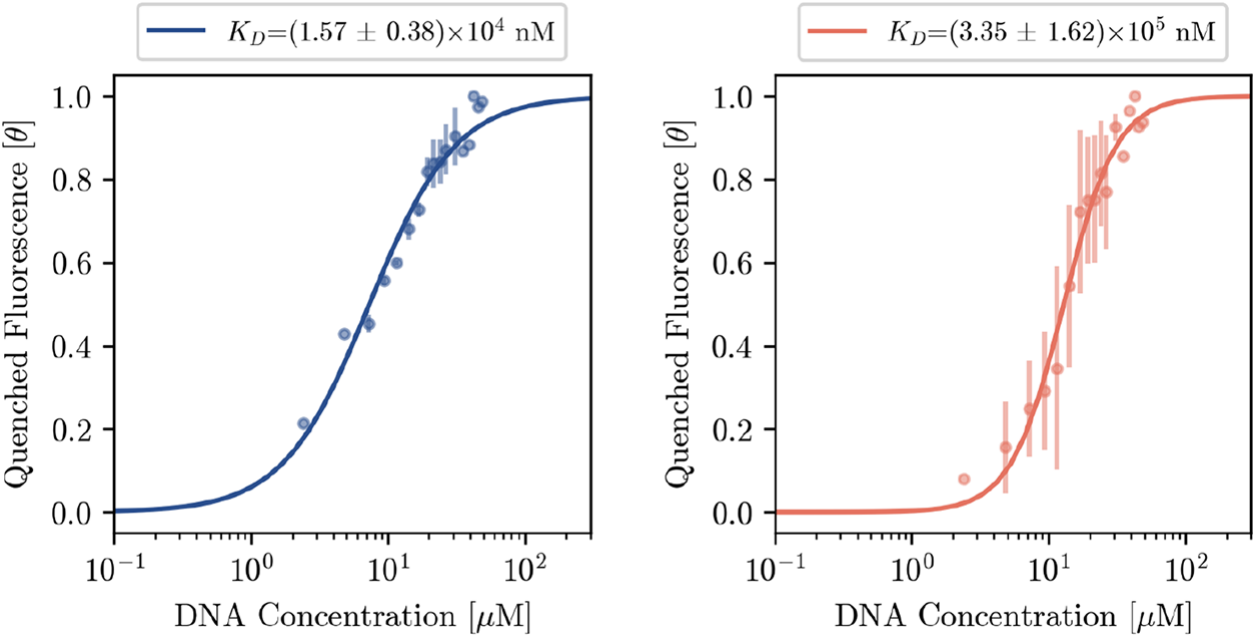
Hill plots for the ApT (left) and GpC (right) of the binding interaction between H-NS and ApT and GpC are shown with the *x*-axis the log concentration of added DNA and *y*-axis the fluorescence intensity fraction θ. Hill coefficients are fitted to *n*_ApT_ = 1.38 ± 0.10 and *n*_GpC_ = 2.27 ± 0.17. The concentration range included in the fits is listed in the Supporting Information.

The change in fluorescence emission of Trp-109 is caused by a change in its environment upon binding DNA. Previous work^67^ showed that the conformation of H-NS does not change upon binding DNA. As the fluorescence emission changes for both nucleotide sequences, it is most likely caused by the negative charge of the DNA coming closer to Trp-109. Molecular dynamics simulations (3 × 1 μs) are performed to analyze interactions between the H-NS DBD and DNA (Figure 3). The 2D density plots show the number of contacts between the QGR motif (residues Glu-112, Gly-113, Arg-114) and DNA, *C*_minor–QGR_, plotted against contacts between the NE1 atom of Trp-109 and the deoxyribose atoms of the DNA backbone *C*_sugar–Trp_, for ApT (left) and GpC (right) sequences. Both plots show that Trp-109 has at least 2 contacts with the DNA backbone when the QGR motif is inserted into the minor groove (indicated by *C*_minor–QGR_ values larger than 25), indicating that the DNA is sufficiently close to alter the fluorescence of Trp-109, regardless of nucleotide sequence. For the ApT sequence, a high density cluster in the upper right quadrant indicates stable interactions between the QGR motif and Trp-109 with the DNA backbone, showing that there is no dissociation of the protein–DNA complex. In contrast, for the GpC sequence, the contact distribution is more scattered, suggesting less stable interactions due to partial dissociation of the Q112 or R114 residues, which increases the distance between Trp-109 and the DNA. The closer proximity of Trp-109 to the DNA backbone in the ApT sequence explains the observed red shift (∼5 nm) upon increasing the DNA concentration in fluorescence emission. However, while this shift supports the notion of increased H-NS stability on AT-rich DNA, it is important to recognize that other factors, such as DNA conformational changes, may also influence fluorescence emission.

**Figure 3:**
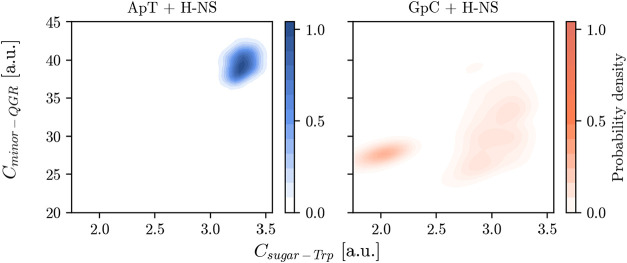
2D probability distributions showing the Trp109 and DNA backbone deoxy ribose contacts with respect to the QGR binding motif contact of H-NS and DNA minor groove acceptors. Left for the ApT sequence (blue) and right for the GpC sequence (red). Probability distributions were computed for 3 × 1 μs of MD simulations for both complexes.

### H-NS Induces Structural Adaptation in DNA Upon Binding

We analyzed the structure of DNA with and without H-NS bound using MD trajectories followed by circular dichroism (CD) spectroscopy. MD simulations provided detailed insights into the local changes in DNA geometry upon H-NS binding, while CD spectroscopy offered a broader perspective on global DNA conformational changes, allowing us to characterize sequence-dependent responses in ApT (AT-rich) and GpC (GC-rich) DNA.

Using the MD trajectories, we analyzed the deoxyribose sugar pucker and glycosidic torsion angles (χ) of each base to evaluate the conformational changes in DNA both in the presence and absence of H-NS. These parameters indicate A-like DNA features (see the Supporting Information for the definitions used). Figure 4A shows that in the absence of H-NS, both ApT and GpC sequences primarily exhibit the South pucker (C2′-endo), typical of B-DNA. However, in the presence of H-NS bound to the minor groove, significant changes occur in the ApT system: a shift toward the North pucker appears at the sixth base step (A-T) where H-NS binds to the DNA, with *P*_North_ reaching 0.95, indicating an A-DNA-like configuration. Supporting Figure S1B shows the difference in conformation between South and North pucker of the deoxribose ring. The neighboring base steps also show partial transitions in the ring puckering, suggesting local structural adaptations. Glycosidic torsion analysis further confirms the presence of A-DNA characteristics in the ApT system with H-NS bound. Although both systems initially showed χ_high–anti_ angles typical of B-DNA,^6^ H-NS binding shifts the ApT system toward the χ_anti_ configuration, indicating an intermediate conformational A/B state. In contrast, the GpC system exhibits minimal changes in the sugar pucker and glycosidic angles, maintaining the canonical B-DNA conformation.

**Figure 4:**
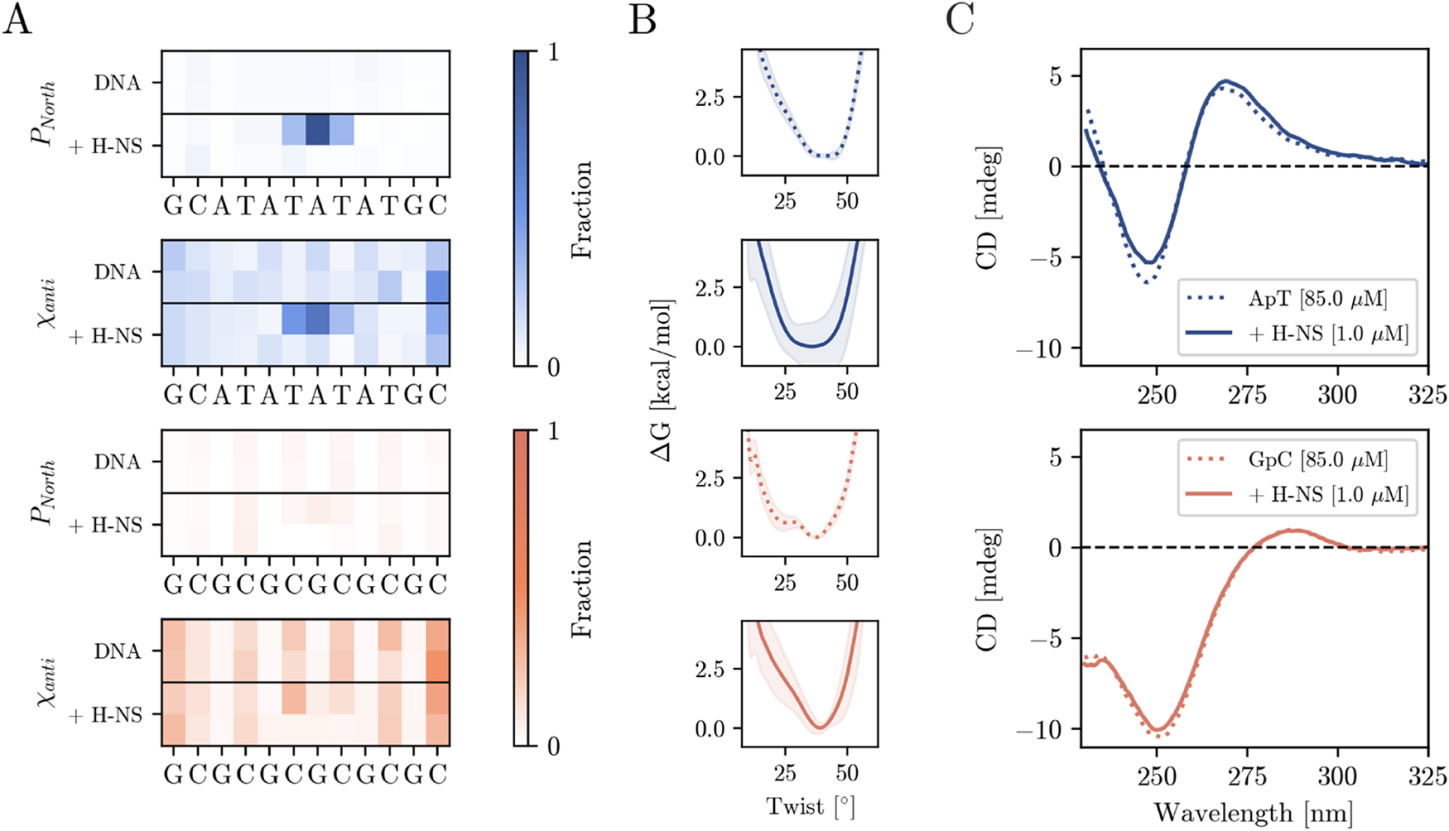
(A) Fractions of the sugar phase in North configuration and χ torsion in anti configuraton for each base pair of the ApT and GpT system with H-NS bound (+ H-NS) and unbound (DNA) based on the analysis of the MD simulations. The Supporting Information contains a detailed explanation of the differences in structure of B- and A-DNA. (B) Free energy profiles of the central base pair’s twist obtained using metadynamics simulations of the ApT (blue) and GpT (orange) system with H-NS bound (solid lines) and unbound (dotted lines). (C) Circular Dichroism spectra of ApT (top) GpC sequence (bottom) in the presence of 1 μM of DBD-H-NS in 20 mM Tris-HCl (pH = 8.0) + 50 mM KCl at 298 K (solid lines) and without (dotted lines). Concentration of all sequences are maintained at 85 μM per base.

We explored the influence of H-NS on the local geometry of DNA using rigid base parameters,^68^ which treats each DNA base as a rigid entity with defined spatial positioning and orientation. As shown in Supporting Figure S6, H-NS binding induces significant increases in the buckle (from 1.6 to 7.3°) and opening angles (from 4.6 to 7.3°) in the ApT sequence, reflecting local DNA bending and unwinding. Furthermore, the twist angle decreases from 33.4 to 29.8°, while the roll increases by 5.8°, suggesting a conformational shift toward an intermediate state A/B. Interestingly, the slide parameter, often used to distinguish A-DNA from B-DNA, shows minimal changes, supporting the idea of a partial rather than a complete B-to-A transition. In contrast, the GpC system remains stable in terms of the parameters of the structural rigid base pair after H-NS binding, exhibiting minimal changes in the twist (stable around 35°), roll, and rise angles. A slight shift in the stagger parameter and a bimodal distribution in the shift angle hint at subtle structural alterations, but these fail to indicate significant deviations from the B-DNA conformation. DNA curvature analysis (Supporting Figure S7) shows that while both sequences exhibit baseline flexibility, the curvature increases by approximately 25% in ApT DNA when the H-NS binds and the helical axis bends toward the minor groove. The GpC system, on the other hand, shows only negligible shifts in flexibility. MD simulations also reveal changes in DNA groove widths upon H-NS binding, highlighting additional indicators of A-like structural features (Supporting Figure S8). For the ApT sequence, H-NS binding causes a widening of the minor groove by approximately 0.4 nm, which is consistent with A-DNA-like characteristics. The major groove displays an asymmetric response, with widening toward the 5′end and narrowing at the 3′ end, indicating a nonuniform conformational shift. One explanation for the higher flexibility of the ApT sequence is that the GpC sequence most likely has a higher melting temperature than ApT, as G-C basepairs have three hydrogen bonds instead of two. Even though no interbase hydrogen bond breaks upon H-NS binding, the increased flexibility of the ApT sequence may be caused by the weaker interaction between the bases.

To investigate and quantify the local changes observed in DNA due to H-NS binding, we performed metadynamics simulations^40^ to study the effects of under and overtwisting of DNA. The metadynamics approach enhances sampling in a molecular dynamics simulation by adding small repulsive potentials along a collective variable at fixed time intervals, slowly pushing the system out of free energy minima. This history-dependent bias potential grows until the system can diffuse freely along the collective variable, after which the bias potential has become the negative free energy along the collective variable. We used the Rigid Base Biasing for Nucleic Acids (RBB-NA) algorithm^65^ to apply a history-dependent bias potential on the twist of the central base step. Notably, this is the base step in which we observe an A-DNA-like configuration in the backbone of the ApT DNA when H-NS binds. In the microsecond-long MD simulations, diffusion along this twist occurs in a much smaller range, as illustrated by time traces in the Supporting Information, Figure S3. Furthermore, ApT with H-NS shows transitions between lower (around 25°) and higher (around 45°) values for the twist, yet only a few, providing poor statistics on this transition. Figure 4B illustrates the free energy profiles of the twist. The shift in the free-energy minimum of approximately 5.8° and the increase in the free-energy valley’s width in the ApT sequence with H-NS bound (compared to that of the unbound state) confirm that DNA adopts a more undertwisted conformation. In contrast, the GpC sequence with and without the H-NS bound shows a much narrower profile, with a stable minimum around 38°. Like the ApT sequence, the GpC sequence exhibits harmonic behavior around the minimum free energy. However, bare DNA deviates from the harmonic free energy profile under high under-twisting, with twist values below 25. In the overtwisted regime, the profile conforms to the expected quadratic shape based on elastic twistable worm-like chain models,^69,70^ until reaching about 30° of twist, at which point a sudden transition in the landscape occurs. Closer inspection of the trajectories reveals that this transition results from partial hydrogen bond breaking, which leads to the opening of one of the bases. Previous studies using umbrella sampling^65^ have shown similar transitions under conditions of significant deviations from equilibrium twist values.

In addition, we measured the correlation between the twists of adjacent base steps, observing a consistent negative correlation coefficient between neighboring base steps (see Supporting Figure S9). Our findings align with the literature on nonlocal couplings in DNA mechanics. The rigid base parameters of DNA, such as twist, roll and tilt, exhibit correlations on multiple nucleotides, demonstrating couplings between distal sites.^71–73^ Recent umbrella sampling simulations have shown that localized perturbations in DNA, such as twists, propagate as oscillating decays along neighboring nucleotides, consistent with nonlocal elasticity models.^65,69,70^ With H-NS bound, the anticorrelation became stronger, reaching approximately −0.73 in the ApT sequence and GpC sequence, compared to −0.55 and −0.58, respectively, in the unbound state. Interestingly, our results demonstrate that H-NS binding is able to amplify anticorrelations in the mechanical twist coupling in both GpC and ApT sequences. Furthermore, our findings show that H-NS binding stabilizes the DNA in the GpC sequence by preserving its twist conformation, whereas in the ApT sequence, the DNA becomes more flexible because of a broadened free-energy profile and under-twisting.

To validate the structural variations observed in the MD simulations, we used CD spectroscopy to detect global DNA conformational changes. CD spectra of DNA show characteristic peaks at approximately 245 nm (negative) and 275 nm (positive), see Figures 4C and S11B. The band at 245 nm is attributed to the inherent chirality in the helical structure, while the 275 nm band arises from the noncentrosymmetric stacking of nucleobase pairs.^74^ Upon H-NS binding, the CD spectra for ApT DNA indicate a decrease in the intensities of these characteristic bands (Figure 4C), suggesting a transition from B-DNA to an A-like conformation. Also, a slight redshift of 1 nm occurs at 275 nm for the ApT system with H-NS bound, indicating an increase in the stacking of the nucleobases. This result aligns with the MD observations of an increase in sugar pucker and altered glycosidic torsion angles in the ApT sequence. In contrast, the CD spectra of GC-rich DNA display minimal changes upon H-NS binding, suggesting that the helical structure remained largely in the B-DNA form. The stability of the GpC sequence in the CD data supports the MD findings of minimal conformational shifts.

### Implications for H-NS Function

The structural changes we observe in DNA curvature, minor groove widening, and reduced twist upon H-NS binding suggest that H-NS enhances the flexibility of AT-rich regions, potentially facilitating regulatory interactions such as protein binding. This effect is reminiscent of the structural changes seen in the TATA box upon binding by the TATA box binding protein (TBP). In the TBP-DNA complex (e.g., 1QNE), TBP binds to the minor groove, causing significant bending, minor groove widening, and reduced helical twist.^75,76^ Furthermore, the sequence-specific changes observed in the ApT and GpC regions due to H-NS binding align with molecular dynamics simulations using umbrella sampling, which revealed that the TATA sequences exhibit the highest flexibility of twist, while the AATT and GCGC sequences are among the stiffest.^77^ This underscores the adaptability of flexible sequences like ApT to conformational changes, compared to the greater rigidity of sequences like GpC. Similarly, H-NS-induced changes in ApT sequences suggest a shift toward a flexible A/B intermediate state, enhancing local DNA plasticity. In contrast, GpC sequences remain structurally stable, highlighting sequence-specific effects associated with H-NS DNA binding. This suggests that H-NS, like TBP, may use induced DNA deformations to facilitate regulatory processes. These results illuminate the broader concept of how DNA-binding proteins may take advantage of sequence-specific structural adaptability to modulate chromatin dynamics and gene regulation. Understanding these mechanisms not only deepens our knowledge of the organization of the bacterial genome but also provides a conceptual framework for studying similar processes in other organisms.

The choice of force field significantly impacts the accuracy of MD simulations. Although the results from the steered MD^32,35^ align with experimental trends, the AMBER bsc1 force field used in this study is known to overstabilize B-DNA and underestimate the stability of A-like conformations.^76,78^ This limitation may have led to an underestimation of the extent and stability of A-like features in DNA bound to H-NS. Recent advances, such as the OL24 force field,^7^ which better stabilizes the North sugar pucker in A-DNA, could improve the modeling of A/B equilibria. OL24 also offers improved accuracy in representing DNA/RNA hybrids and protein–DNA complexes, suggesting that future simulations using such refined force fields may provide deeper insights into the structural transitions observed in this study.

To further explore H-NS binding to DNA, we used calf thymus DNA (ct-DNA), which is much larger than the 12-mer constructs ApT and GpC, and 4′,6-diamidino-2-phenylindole (DAPI) as a known minor groove to AT-rich sequences.^79^ Steady-state and time-resolved fluorescence spectroscopy was utilized to measure the fluorescence decay of DAPI in different DNA environments, see Supporting Figures S10 and S11A. These measurements allow for the detection of changes in the DNA structure upon interaction with H-NS. The reason is that DAPI’s fluorescence decay is sensitive to its local environment, including the DNA sequence to which it binds, and thus changes in the fluorescence lifetimes can indicate alterations in the conformation or dynamics of DNA. Supporting Figure S11A shows the change in fluoresence of DAPI upon the addition of H-NS. Adding the protein alters the emission and excitation spectra, indicating that either H-NS is replacing DAPI from AT sites on the DNA or that the secondary structure of the DNA is altered. This modification may affect the hydration of the DAPI microenvironment upon more DBD binding to DNA. The results on the short DNA constructs showed that there is cooperativity in the binding of H-NS and DNA. Consequently, the continuous change in emission and excitation upon H-NS addition to ct-DNA can also be attributed to the protein–DNA cooperative binding. CD spectroscopy of ct-DNA shows the two distinctive peaks associated with ct-DNA, specifically a negative band at approximately 245 nm and a positive band at approximately 275 nm, see Supporting Figure S11B. Upon the addition of H-NS to ct-DNA, both bands exhibited an increase in their CD values. This observation suggests that the DBD influences the helical conformation of DNA as well as the stacking arrangement of bases in the secondary structure.

Although our molecular dynamics simulations were limited to relatively small DNA fragments, the experimental observations on ct-DNA indicate that multiple H-NS DBDs can bind simultaneously to the DNA molecule, opening the possibility of DNA-mediated allosteric interactions. In this context, binding of an H-NS DBD could influence the affinity and conformation of neighboring DNA regions through mechanisms that propagate binding-induced perturbations along the DNA strand through distal couplings between DNA base pairs.^80^ Our fluorescence and CD spectroscopy experiments with ct-DNA, which indicated cooperative binding effects, likely reflect the influence of multiple H-NS DBDs acting on the DNA, resulting in sequence-dependent stabilization or destabilization effects. These allosteric influences could lead to significant alterations in the DNA conformation, contributing to the observed B-to-A form transition, particularly in AT-rich regions. Such cooperative binding might explain the more pronounced conformational changes in ct-DNA compared to those of isolated DNA fragments, highlighting the potential impact of large-scale allosteric effects on H-NS-DNA interactions. However, this interpretation remains speculative as direct evidence for DNA-mediated allosteric effects on H-NS binding is limited. Nevertheless, related studies provide notable parallels and insights. In Bacillus subtilis, DNA-mediated allostery arises from changes in curvature and spacer-dependent mechanical tension, with the cooperativity of transcription factors significantly influenced by groove width deformations.^81^ Cryo-EM and smFRET experiments showed that ComK binding led to a widening of the minor groove in AT-rich binding sites, which subsequently affected the width of the minor groove elsewhere depending on the length of the linker DNA, reflecting how DNA mechanics can propagate structural changes. These findings allow us to speculate about allosteric effects in DNA as induced by H-NS, causing minor groove widening and increased curvature in AT-rich DNA. Our fluorescence and CD spectroscopy experiments suggest the potential for mild cooperative interactions mediated by structural perturbations in DNA, particularly in AT-rich regions. However, more direct investigations, such as cryo-EM, smFRET, or single-molecule dissociation assays, are required to confirm whether DNA mechanics similarly modulate H-NS cooperativity through long-range allosteric effects.

The conformational changes in DNA, as observed in H-NS interactions with AT-rich sequences, open several questions for further investigation. First, expanding the use of advanced sampling techniques, such as the RBB-NA algorithm, to explore other DNA-binding proteins could provide a more comprehensive understanding of how different sequence contexts influence protein–DNA binding specificity and stability. Investigating whether other nucleoid-associated proteins (NAPs) induce similar or distinct DNA conformational changes could aid in understanding the broader principles governing the organization of bacterial chromatin. In particular, many NAPs preferentially bind AT-rich DNA, a characteristic central to their roles in chromatin organization. It is worth exploring how NAP binding influences other proteins that interact with DNA, even without direct protein–protein interactions, potentially through changes in DNA topology or conformation. These dynamics, particularly relevant on longer DNA fragments, could reveal cooperative or allosteric effects shaping binding and chromatin structure. Furthermore, extending the study to longer DNA fragments with more H-NS DBD bound would be valuable to assess whether and how allosteric or cooperative effects are present in H-NS and similar proteins and affect regulatory elements in vivo or in vitro. This could shed light on how sequence-dependent flexibility contributes to the formation of higher-order nucleoprotein structures, ultimately affecting gene regulation. Another interesting direction would be to investigate the impact of H-NS binding on DNA mechanical properties under various environmental conditions, such as changes in ionic strength, temperature, or supercoiling. These factors can modulate DNA conformation and binding affinity, potentially altering the protein’s ability to induce sequence-specific structural transitions. This approach could be combined with single-molecule techniques, such as magnetic or optical tweezers, to directly observe H-NS-induced twist and bending changes in real time. Such insights could further explain how different NAPs dynamically interact to regulate chromatin states under varying physiological conditions.

## Conclusions

The intrinsic fluorescence of Trp-109 in H-NS is quenched when its DNA binding domain binds to DNA, allowing for an estimation of dissociation constants. Titration of H-NS with ApT or GpC dsDNA showed that the DNA binding domain of H-NS has a higher dissociation free energy of around 30% for ApT compared to GpC, matching the predicted difference of 40% in favor of the ApT sequence. Our results demonstrate that H-NS binding induces sequence-specific conformational changes in DNA, with AT-rich regions showing significantly greater variability in structure compared to GC-rich sequences. Molecular dynamics simulations revealed that in the presence of H-NS, AT-rich ApT sequences undergo undertwisting, minor groove widening, and a local shift toward an intermediate A/B state, as characterized by local increases in the pucker of deoxyribose groups and shifts in glycosidic torsion angles. These changes were marked by increases in flexibility-related parameters such as buckle and roll, while GC-rich GpC sequences maintained their canonical B-DNA conformation with minimal structural perturbations. Metadynamics simulations further supported these findings, showing that H-NS binding broadens the free energy landscape of twist in ApT sequences, indicating increased flexibility. CD spectroscopy confirmed these trends, with AT-rich DNA exhibiting a shift toward A-like characteristics, while GC-rich DNA remained predominantly in the B-DNA form. Together, these results highlight the preferential binding and conformational modulation of AT-rich DNA by H-NS, driven by its intrinsic flexibility and adaptability to protein-induced structural changes.

## Supplementary Material



## Data Availability

Data set: 10.6084/m9.figshare.c.7581857.v1.

## Notes

Text and figures; adaptation of DNA to protein binding revealed by spectroscopy and molecular simulation (PDF)
