# Supplementary material for: Adaptation of DNA to Protein Binding Revealed by Spectroscopy and Molecular Simulation
Source: J Phys Chem B. 2025 May 28;129(23):5653–63. doi: 10.1021/acs.jpcb.5c00189 (PMC12169650; doi:10.1021/acs.jpcb.5c00189)
Supplement: Supplementary file 1 [file jp5c00189_si_001.pdf]

Supporting Information: Adaptation of DNA to Protein Binding Revealed by Spectroscopy and Molecular Simulation

Thor van Heesch,[†] Sudhanshu Sharma,[‡] Bert van Erp,[¶,§,‖] Alberto Pérez de Alba Ortíz,[†,⊥] Remus T. Dame*,[*,¶,#,‖] Jocelyne Vreede*,[*,†] and Krishna Gavvala[*,‡]

†*Van 't Hoff Institute of Molecular Sciences, University of Amsterdam, Science Park 904, 1098 XH Amsterdam, The Netherlands*

‡*Department of Chemistry, Indian Institute of Technology Hyderabad, Kandi, Sangareddy, Telangana 502284, India*

¶*Leiden Institute of Chemistry, Einsteinweg 55, 2333 CC Leiden, The Netherlands.*

§*Centre for Microbial Cell Biology, Leiden University, Einsteinweg 55, 2333CC Leiden, The Netherlands*

‖*Centre for Interdisciplinary Genome Research, Leiden University, Einsteinweg 55, 2333 CC Leiden, The Netherlands*

⊥*Informatics Institute, University of Amsterdam, Science Park 904, 1098 XH Amsterdam, The Netherlands*

#*Centre for Microbial Cell Biology, Leiden University, Einsteinweg 55, 2333 CC, Leiden, The Netherlands*

E-mail: rtdame@chem.leidenuniv.nl; j.vreede@uva.nl; kgavvala@chy.iith.ac.in

Structural Analysis

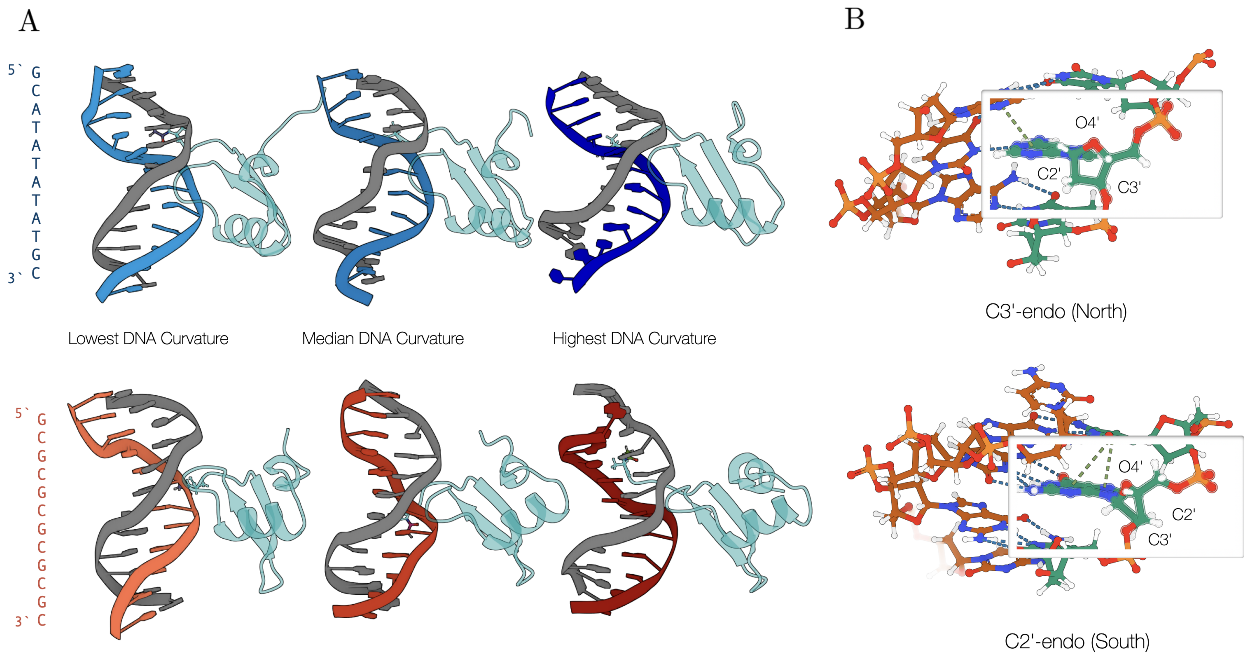

.

Supplementary Figure S1: **A)** Molecular visualization of H-NS (teal) bound to the minor groove of the ApT (left, blue) and GpC (right, orange/red) DNA sequences. From left to right, the structures are derived from the lowest, median and highest curvature values of the DNA, computed from molecular dynamics trajectories. **B)** The left panel presents a zoomed-in molecular representation of the sugar puckering in the C3'-endo (North) conformation at the central base pair step of the ApT DNA, obtained from molecular dynamics trajectories. The right panel depicts the C2'-endo (South) conformation at the central base pair step of the GpC sequence. In both cases, H-NS is bound (not shown). Visualizations were rendered using Mol*Viewer.[1]

Characterization of A-form v B-form DNA

Sugar pucker was considered North (N) if P was less than 90 or greater than 270, otherwise it was considered South (S). The glycosidic angle was considered anti in the interval between 120 and 227.5 ° and high-anti when it was greater than 227.5 (syn otherwise; syn populations were negligible and are not reported). The value of 227.5 was chosen as a midpoint between the v values for pure B-form (BB00) and pure A-form (AA00) according to Cerny et al.

(2020).[2] This distinction is arbitrary, and it should be kept in mind that the v distributions similar to those of A and B are relatively wide and may overlap; therefore, a clear and unambiguous distinction is not possible. For more background on definitions, see Methods and .

Torsion Angle χ about the glycosidic bond

The chi (χ) torsion angle is a key structural parameter in DNA, describing the orientation of the glycosidic bond between sugar (deoxyribose) and the base. In canonical DNA nucleosides, χ is defined for pyrimidines (C, T, U) as $O4' - C1' - N1 - C2$ and for purines (A, G) as $O4' - C1' - N9 - C4$. This angle provides critical information on the spatial arrangement and flexibility of nucleobases within the DNA helix. Furthermore, the angle χ defines the rotation of the base relative to the sugar, indicating whether the base is in an anti- or syn conformation. For B-DNA, the base is oriented away from the sugar, which is the most common conformation in B-DNA corresponding to the anti conformation. In A-DNA, the angle χ still adopts the anti conformation for all bases, similar to that of B-DNA. However, the exact χ values for A-DNA tend to be slightly less negative, generally ranging between $-100°$ to $-120°$, compared to the more negative range seen in B-DNA (around $-120°$ to $-150°$). This less negative χ value results in a different glycosidic bond orientation.

Definition and Computation of the Sugar Phase Angle in DNA

The sugar ring in DNA (and RNA) can adopt multiple puckered conformations, the most prevalent being the C3′-endo (A-DNA or RNA) and C2′-endo (B-DNA) forms see Supplementary Figure S1 for snapshots. These puckers are critical in defining the overall helical structure of DNA, as they influence the distance and orientation between neighboring phosphorus (P) atoms in the sugar-phosphate backbone, thereby distinguishing A- and B-form DNA conformations.

To describe the conformation of the five-membered sugar ring in DNA, we analyze the

five endocyclic torsion angles:

- ν_0: $C4' - O4' - C1' - C2'$
- ν_1: $O4' - C1' - C2' - C3'$
- ν_2: $C1' - C2' - C3' - C4'$
- ν_3: $C2' - C3' - C4' - O4'$
- ν_4: $C3' - C4' - O4' - C1'$

These torsion angles describe the spatial arrangement of atoms around the sugar ring and are typically calculated using molecular modeling software. However, due to the constraints of the five-membered ring, the conformation can be more efficiently characterized using two parameters: the amplitude (τ_m) and the phase angle (P), representing the puckering state of the sugar ring along its pseudorotational pathway. A widely adopted method to compute the sugar phase angle P and amplitude τ_m is based on the work of Altona & Sundaralingam (1972).[3] In this method, the relationship between the five torsion angles ν_0 to ν_4 and the phase angle P is described by a pseudorotational model. This model effectively tracks the puckering motion of the sugar ring as it oscillates through different conformations. The phase angle P and the amplitude τ_m are computed as follows:

$$P_{const} = \sin\left(\frac{\pi}{5}\right) + \sin\left(\frac{2\pi}{5}\right) \approx 1.5388$$

$$P_0 = \arctan\left(\frac{\nu_4 + \nu_1 - \nu_3 - \nu_0}{2 \cdot \nu_2 \cdot P_{const}}\right)$$

Here, P_0 is the phase angle in radians. To convert P_0 to degrees, we have:

$$P = \frac{180}{\pi} \times P_0$$

If P is negative, add 360 to obtain a positive phase angle. The amplitude τ_m is then calculated as follows:

$$\tau_m = \frac{\nu_2}{\cos(P_0)}$$

Protein-DNA contacts

The minor groove of dsDNA contains mainly hydrogen bond acceptors, except guanine, and the DNA binding motif in H-NS contains mainly hydrogen bond donors. Previous work has identified a quantitative descriptor to follow the interaction between DNA and H-NS by counting the number of contacts between hydrogen bond acceptors in the minor groove of DNA, labeled i, and hydrogen bond donors in the QGR motif of H-NS, labeled j.[4] For each pair ij, we define a contact c_{ij} with the expression:

$$c_{ij} = \begin{cases} 1, & \text{if } (r_{ij} - d_0) < 0, \\ \dfrac{1 - \left(\dfrac{r_{ij} - d_0}{r_0}\right)^{nn}}{1 - \left(\dfrac{r_{ij} - d_0}{r_0}\right)^{mm}}, & \text{if } (r_{ij} - d_0) \geq 0, \end{cases} \tag{1}$$

where r_{ij} is the distance between atom i and atom j, located in the DNA and H-NS, respectively. The parameters $r_0 = 0.4\ nm$, $d_0 = 0.25\ nm$, $nn = 2$, $mm = 4$ have been chosen such that to count contacts at hydrogen bond distance (< 0.35 nm) as 1 and contacts at 0.7 nm as 0.5. This provides a smooth and descriptive function that can discriminate between the different binding modes. Summing all contacts for all pairs results in the contact map parameter $C_{minor-QGR}$:

$$C_{minor-QGR} = \sum_{j=1}^{N_{H-NS}} \sum_{i=1}^{N_{DNA}} c_{ij} \tag{2}$$

where N_{DNA} and N_{H-NS} are the number of interaction sites in the DNA and in H-NS. In this contact map, hydrogen bond donors in Q112, G113 and R114 have been included. In addition, we calculated in the same manner the number of contacts between the nitrogen atom in W109 (NE1) and one of the carbon atoms (C3$'$) in the deoxyribose groups of the DNA backbone (referred to as $C_{sugar-Trp}$).

Additional Metadynamics Analysis

In our study of all systems, we examine the twist to ensure that the dynamics have progressed to the 'free diffusion' stage along the bias coordinate. This indicates an adequate approximation of the free energy profile, which usually occurs within 10 ns of biasing. Occasionally, the DNA opened more than one base pair and did not restore its complementary base pair afterward; such data were removed and not included in the averaging process. Supplementary Figure S3 illustrates the specific metadynamics runs for each system that were considered when averaging to derive the free energy profiles concerning the central base step twist. The transparent time traces are omitted from further analysis.

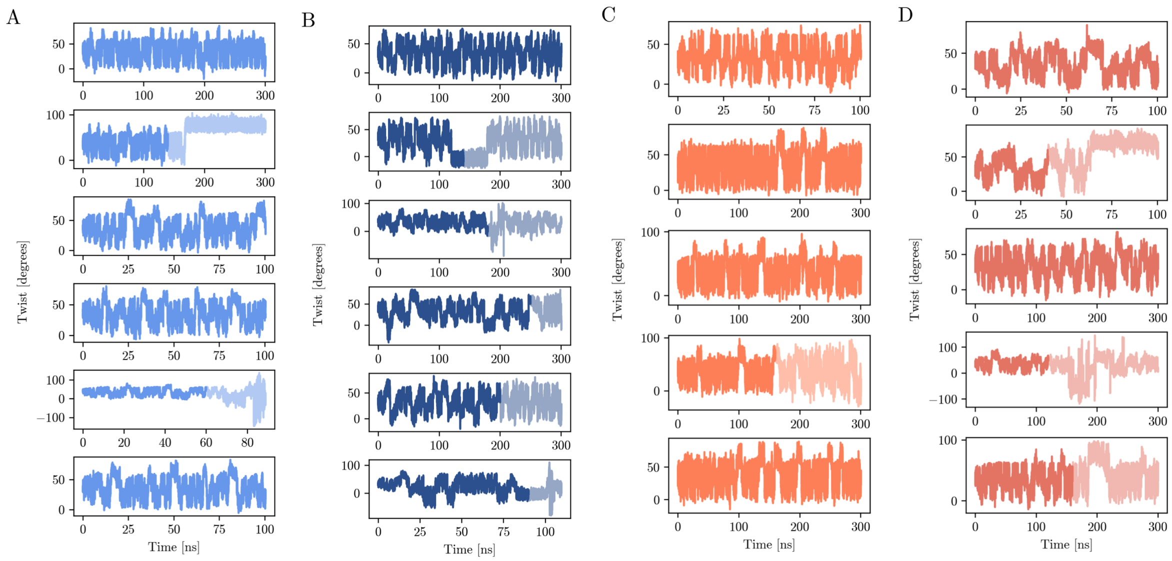

Supplementary Figure S2: Panels A, B, C, and D depict the sampling of the central base step twist value used as a collective variable during the metadynamics simulations for the ApT sequence both without (A) and with H-NS binding (B), as well as for the GpC sequence both without (C) and with H-NS binding (D). The transparent regions in the time traces represent pruned sections that are excluded from further analysis due to the occurrence of base opening or DNA denaturation from excessive twisting that failed to revert to a stable helix. Panels A, B, C, and D respectively exhibit a total of 426, 99, 205, and 205 crossings within the interval of 10 to 60 degrees of twist.

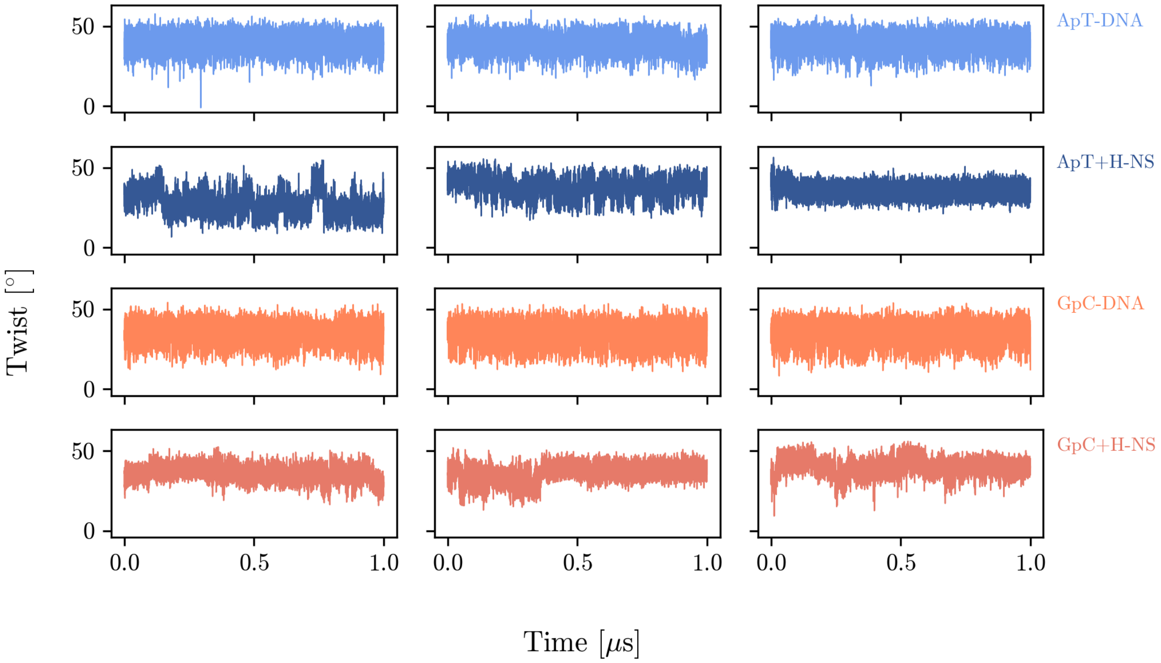

Supplementary Figure S3: Sampling of the central base step twist value along three independent MD simulations of 1 μs of the ApT and GpC dsDNA sequences with and without H-NS (from top to bottom row).

Fluorescence Spectra of H-NS binding to ApT and GpC

While performing the fluorescence lifetime measurements using H-NS intrinsic fluorescence (λ_{ex} = 290 nm) there was a negligible change in the lifetime decay upon adding DNA, indicating a static quenching mechanism, see Supplementary Figure S4-A. Using Trp-109 intrinsic fluorescence quenching, the Stern-Volmer quenching constants (K_{SV}) are 1.8 $\cdot 10^4$ M and 2.1 $\cdot 10^4$ M for ApT and GpC respectively, see Supplementary Figure S4-B. The K_{SV} values for AT and GC-rich DNA are close to each other and both originate from a static quenching mechanism.

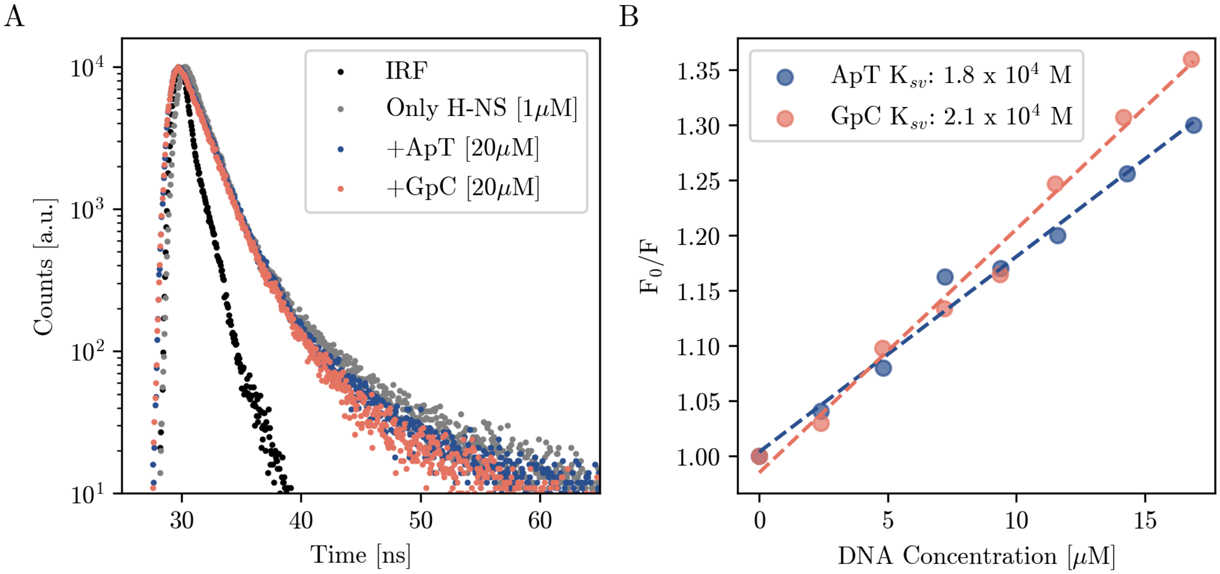

.

Supplementary Figure S4: **A)** Intrinsic fluorescence decay of H-NS (1 μM) in the absence and presence of ApT (20 μM) and GpC (20 μM) DNA. **B)** Stern-Volmer quenching plots for H-NS vs ApT (dark blue) and H-NS vs GpC (dark red) using the following equation $F/F_0 = (1 + K_{SV}[DNA])$ where F_0 and F are the fluorescence intensities of protein in the absence and presence of the quencher concentration [DNA] and K_{SV} is the Stern-Volmer quenching constant.[5] Quenching constants are shown in the figure inset.

We conducted two sets of titration experiments for both the ApT and GpC sequence with 1 μM H-NS DNA binding domain (See Materials Section). Set one we measured a concentration range of 0.0, 4.8, 7.2, 9.36, 11.52, 14.16, 16.8, 19.2, 21.6, 24.0, 26.4, 30.72 μM

DNA (see panel A and B of Supplementary Figure S5). In the second titration, we extended the concentration range to 0.0, 2.4, 4.83, 7.22, 9.38, 11.6, 14.3, 16.9, 19.26, 21.66, 24.0, 26.44, 30.87, 35.0, 38.84, 42.28, 45.6, 48.61 μM DNA (see panel C and D of Supplementary Figure S5).

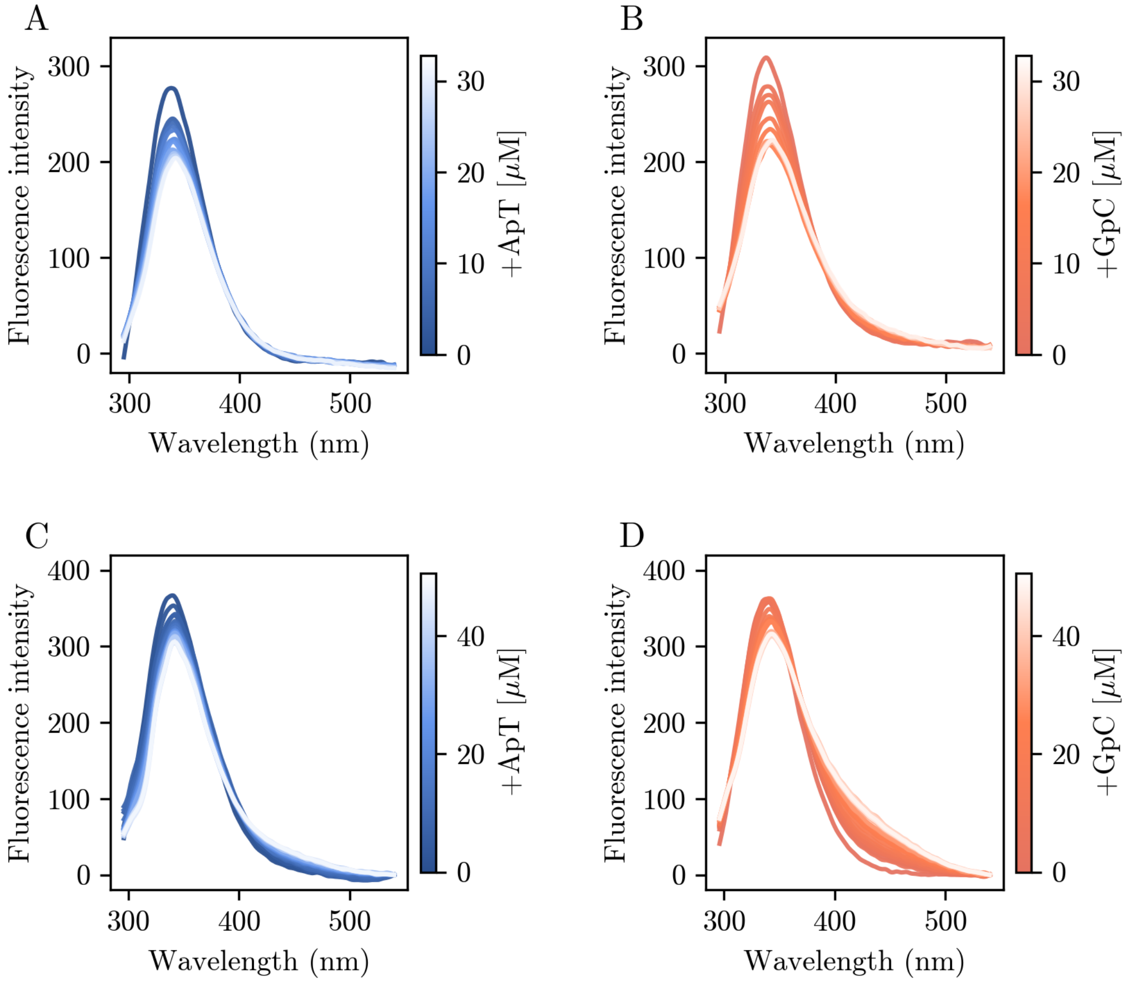

.

Supplementary Figure S5: Emission Spectra (λ_{ex} = 280 nm) of Trp-109 in absence and presence of ApT (blue) and GpC (orange) with 1 μM H-NS.

The binding constants were determined by analyzing the fluorescence-quenching data as shown in Supplementary Figure S5. The procedure involved converting the raw fluorescence

intensity measurements into a normalized fraction of quenched fluorescence, followed by fitting the data to Hill's equation to extract the relevant parameters.[6,7]

The fluorescence intensity (F) was first normalized to account for variations in the maximum observed intensity. The normalized fluorescence intensity (F_{norm}) was calculated using:

$$F_{\text{norm}} = \frac{F}{F_{\text{max}}} \tag{3}$$

where F is the measured fluorescence intensity, and F_{max} is the maximum observed fluorescence intensity in the dataset. The fraction of quenched fluorescence (θ) was then determined from the normalized fluorescence intensity using:

$$\theta = 1 - F_{\text{norm}} \tag{4}$$

Next, θ is further normalized to the maximum fraction of quenching (θ_{max}) using:

$$\theta_{\text{norm}} = \frac{\theta}{\theta_{\text{max}}} \tag{5}$$

This procedure converts the raw fluorescence data into a normalized fraction of quenching, facilitating comparison between different experimental conditions. The resulting θ values were then used to fit Hill's equation, which describes the binding relationship between the concentration of DNA and the fraction of quenched fluorescence:

$$\theta = \frac{[DNA]^n}{K_d + [DNA]^n} \tag{6}$$

Here, n represents the Hill coefficient, K_d is the equilibrium dissociation constant of the ligand–receptor complex,[8] and $[DNA]$ is the concentration of added DNA in μM. The fitting of Hill's equation to the measurements was performed using the `scipy` library in Python.[9] For the concentration range used during the fitting procedure logarithmically spaced values were created from 10^{-1} to the maximum concentration plus 200, spanning 2000 points.

Additional Structural Characterization of MD and meta-dynamics simulations

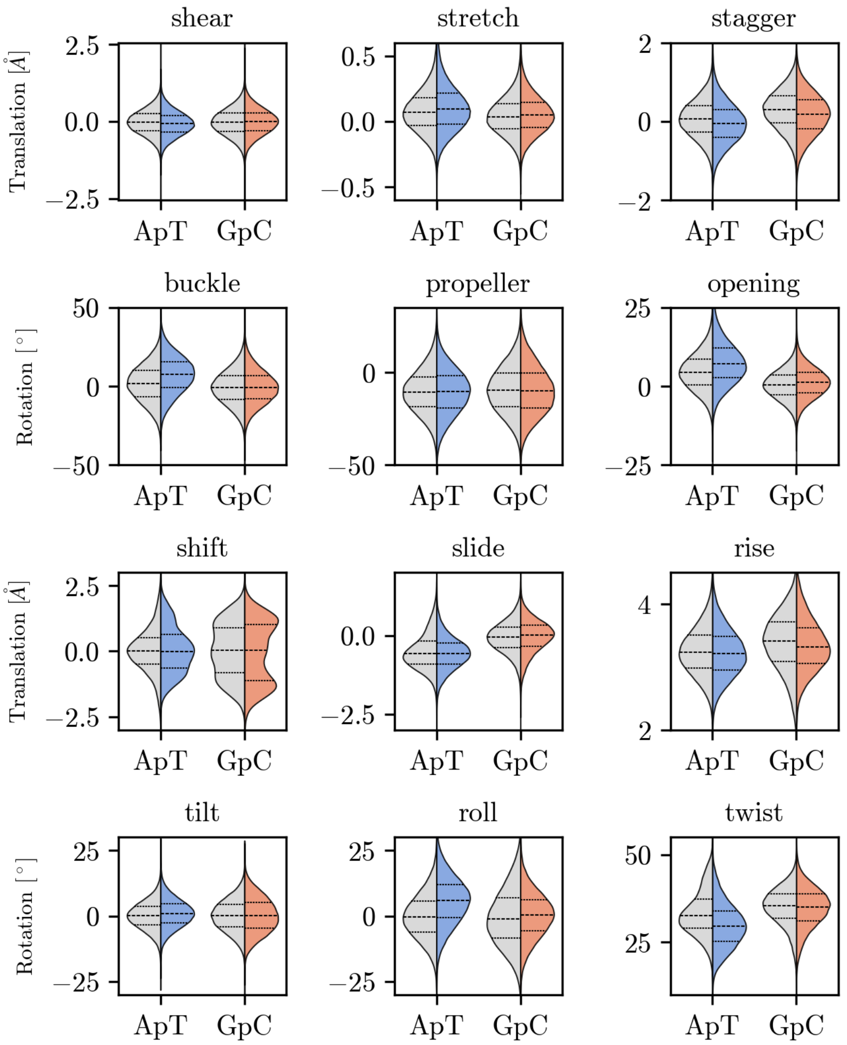

Supplementary Figure S6: Cumulative distributions of base pair parameters for the central base step and its neighboring base pairs (steps 6 to 9) in the ApT and GpC systems, with (blue and red, respectively) and without H-NS (dark grey), were analyzed. The analysis is based on 3x 1 μs of MD simulations solvated in water and 50 mM KCl at 298 K with the initial 500 ns discarded.

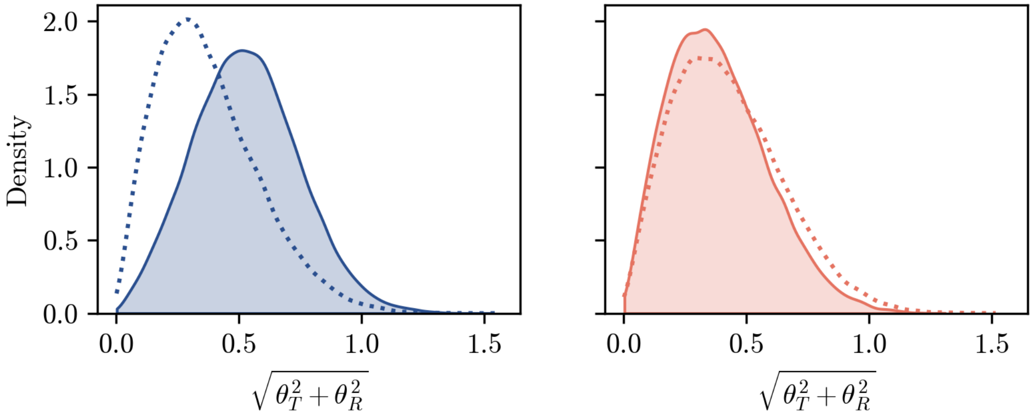

Supplementary Figure S7: For each DNA system (ApT blue and GpC red), we computed the distributions of total magnitude of the curvature using the global tilt (θ_T) and global roll (θ_R) values (which are sums of the projected components of local base step) while adjusting for helical twist in the local base pair steps.[10] The dotted line indicates the total curvature of the bare DNA, and the solid lines with H-NS bound.

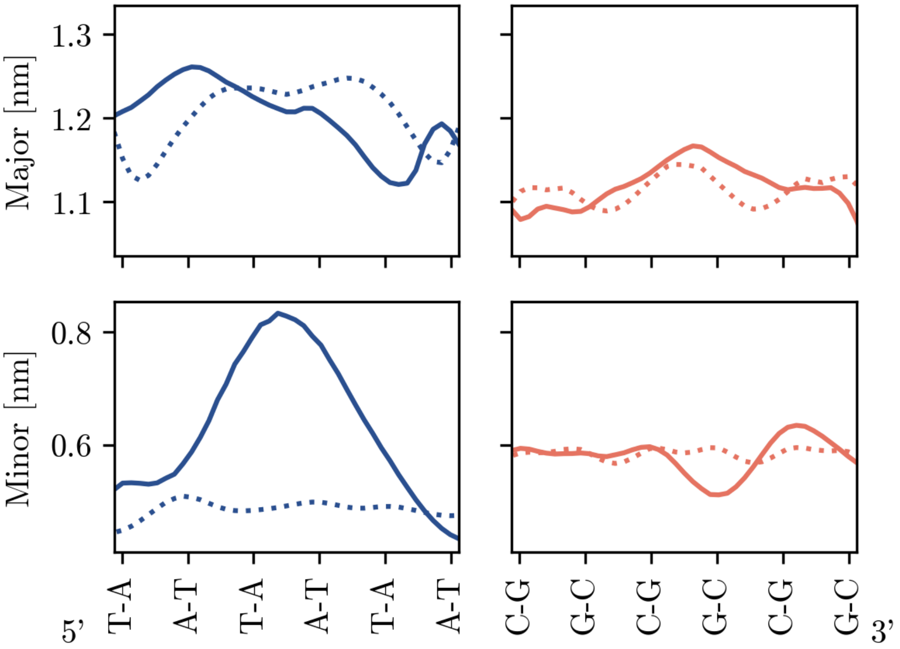

Supplementary Figure S8: Averaged major (top) and minor (bottom) groove widths of the ApT sequence in blue and the GpC sequence in orange with the dotted line the bare DNA without H-NS bound, and the solid line with H-NS bound. Note, the GC-caps are not included in this plot.

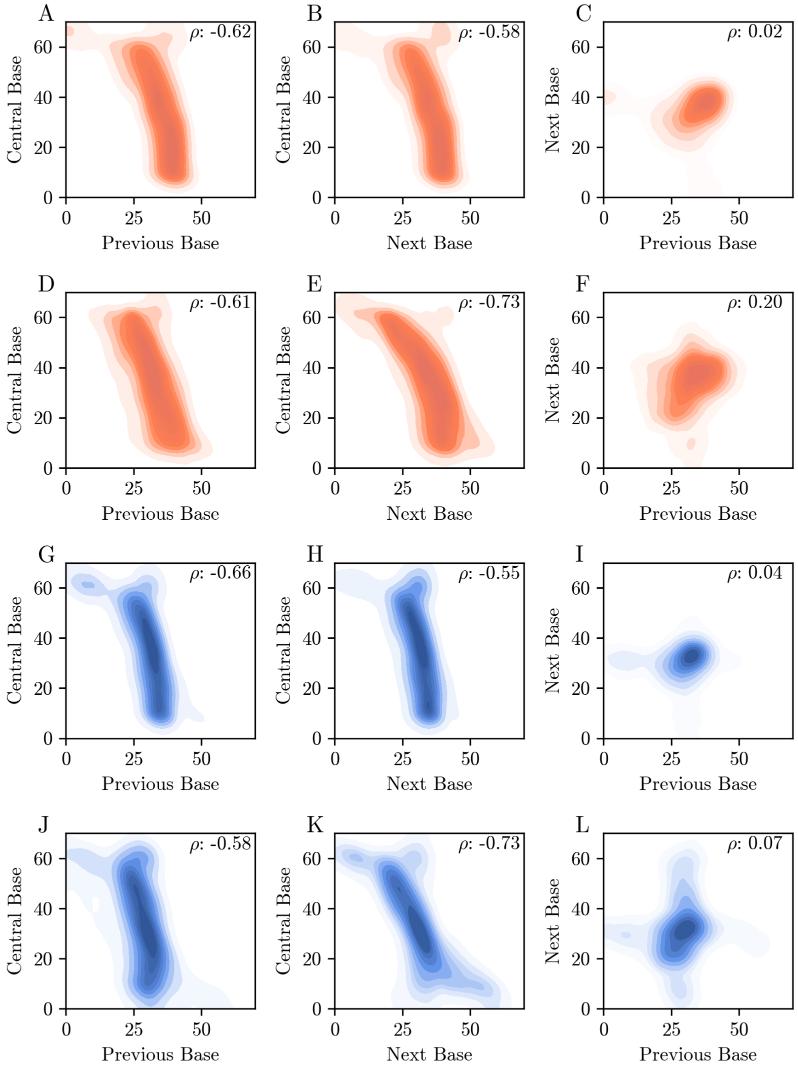

Supplementary Figure S9: Two-dimensional density estimates of the base step twist values were obtained during metadynamics simulations for the central base step and its immediate neighbors. Panels A, B, and C pertain to the GpC system without H-NS binding, while panels D, E, and F relate to the situation where H-NS is bound to the GpC sequence. Panels G, H, and I correspond to the ApT sequence without H-NS binding. Lastly, panels J, K, and L pertain to comparisons between the preceding base and central base, the subsequent base and central base, and between the preceding base and the subsequent base of the ApT sequence with H-NS binding. Each panel also indicates the correlation coefficient between the two evaluated variables. It is noteworthy that only the central base step was subject to bias during the metadynamics simulations.

Validation of H-NS induced structural dynamics in ct-DNA

Calf thymus DNA (ct-DNA) serves as a model system for studying DNA-protein interactions, such as those involving DNA-binding domains (DBD) of proteins such as H-NS. These interactions can affect the stability and structure of DNA, particularly at sites with different sequence compositions (e.g., AT-rich vs. GC-rich regions).[11,12] Given the selective affinity of H-NS for AT-rich sequences, we used time-resolved fluorescence spectroscopy with 4',6-diamidino-2-phenylindole (DAPI), a fluorescent dye that specifically binds to the minor groove of AT-rich DNA,[13] to assess the binding dynamics and structural effects of H-NS on ct-DNA. Steady-state and time-resolved fluorescence spectroscopy was utilized to measure the fluorescence decay of DAPI in different DNA environments, allowing for the measurement of changes in the DNA structure upon interaction with H-NS. The reason is that DAPI's fluorescence decay is sensitive to its local environment, including the DNA sequence to which it binds, and thus changes in the fluorescence lifetimes can indicate alterations in the conformation or dynamics of DNA.

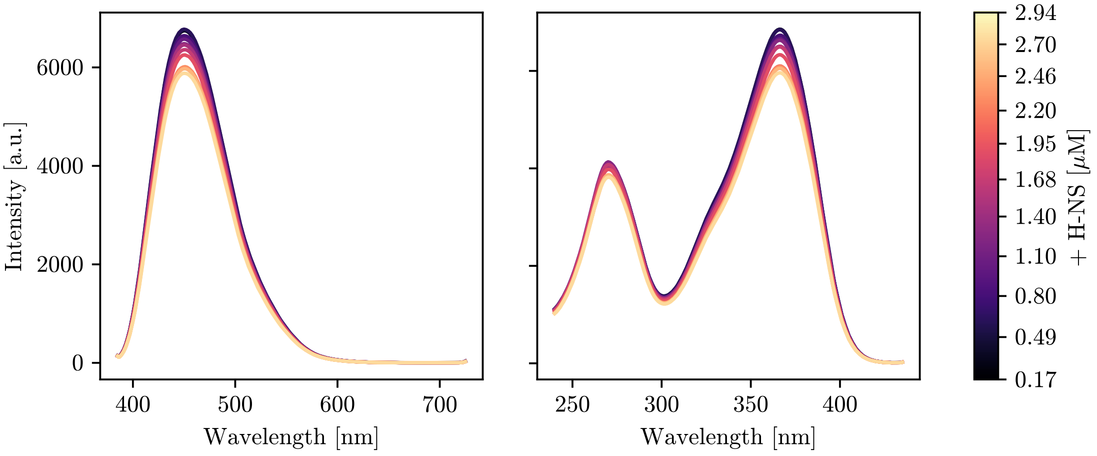

.

Supplementary Figure S10: Emission spectra (λ_{ex} = 370 nm, left) and excitation spectra ((λ_{em} = 450 nm, right) of ct-DNA bound DAPI in presence of different amounts of DBD-H-NS in 20 mMTris-HCl(pH = 8.0) + 50 mM KCl at 298 K.

Supplementary Figure S10 shows changes in the emission spectra (λ_{ex}= 370 nm) of DNA bound DAPI when the DBD of H-NS is introduced. Adding the protein decreased the fluorescence emission at wavelength at 450 nm. The decrease in intensity may indicate that either HNS is replacing DAPI from AT sites on the DNA or that the secondary structure of the DNA is altered. This modification may affect the hydration of the DAPI microenvironment upon more DBD binding to DNA. From the study with the short DNA constructs, we saw that there is cooperativity in the binding of H-NS and DNA. Consequently, the continuous change in emission upon H-NS addition to ct-DNA can also be attributed to the protein-DNA cooperative binding. Supplementary Figure S10-B depicts the excitation spectrum of DNA with DAPI bound, collected at λ_{ex} = 450 nm, corresponding to the different amounts of H-NS DBDs. Similar to the changes in the maxima of the emission spectra, the maximum at 370 nm in the excitation spectra decreased in intensity upon binding of ct-DNA with the DBD. Both emission and excitation spectra indicate there is either replacement of DAPI by H-NS or formation of a ternary complex with the DNA.

Supplementary Figure S11-A illustrates the fluorescence decay characteristics of free DAPI, showing two distinct lifetime components: a shorter component of approximately 0.2 ns and a longer component of 2.9 ns. These components can be attributed to different excited state behaviors of DAPI, which result from a proton transfer process between its indole and amidino nitrogen atoms.[13] Upon interaction with ct-DNA, the longer lifetime component extends to 3.5 ns, a finding that implies the interaction of DAPI with AT-rich regions of the DNA, while the shorter component remains unaltered. This phenomenon is consistent with DAPI's established affinity for AT-rich sequences, thereby distinguishing these regions from GC-rich areas on the basis of variations in fluorescence lifetime. The introduction of H-NS to the DAPI-ct-DNA complex resulted in an increase in the longer lifetime component from 3.5 to 3.7 ns, accompanied by a significant increase in the relative contribution of this component from 0.46 to 0.94. This alteration suggests that H-NS binding induces conformational changes within DNA that affect the local environment of AT-rich

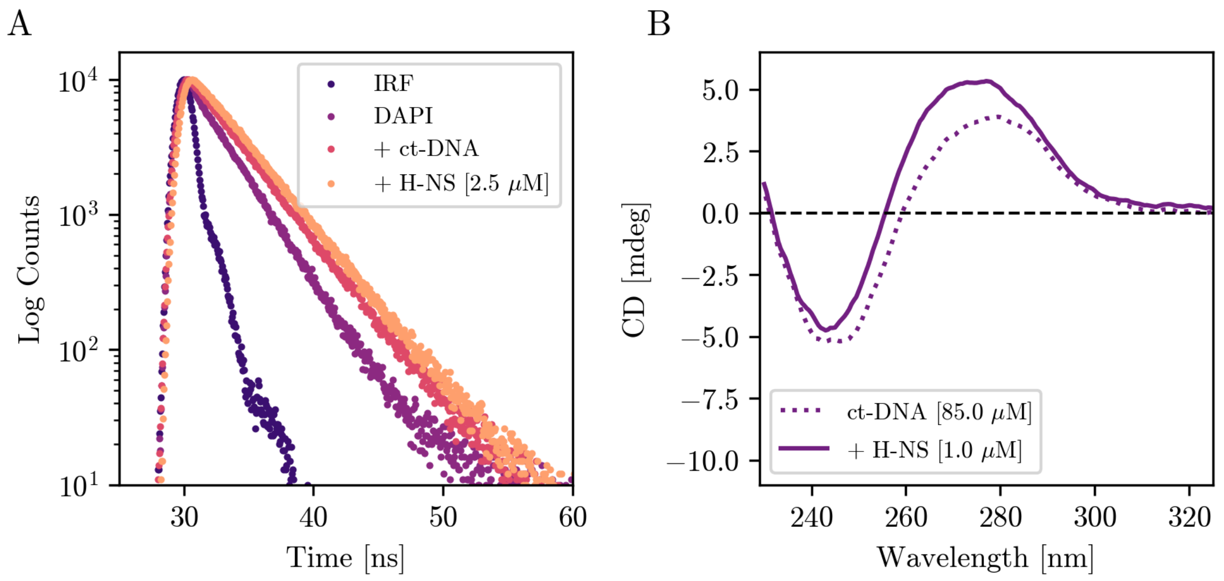

.

Supplementary Figure S11: **A)** Fluorescence decay profile of free DAPI, ct-DNA bound DAPI in absence and presence of (2.5 μM DBD-H-NS in 20 mM Tris-HCl (pH= 8.0)+50 mM KCl at 298 K. (λ_{ex}= 370 nm, λ_{em}= 450 nm) **B)** Circular Dichroism Spectra of ct-DNA sequence in presence of 1 μM of DBD-H-NS in 20 mM Tris-HCl (pH = 8.0) +50 mM KCl at 298 K. Concentration of all sequences are maintained at 85 μM per base.

regions complexed with DAPI, thereby enhancing the inhibition of proton transfer between the indole and amidino groups of DAPI. These findings indicate that H-NS binding does not merely displace DAPI; rather, it induces structural changes that further restrict the solvation environment surrounding DAPI, consequently inhibiting DAPI solvent interactions, as evidenced by the increased fluorescence decay lifetime.

The secondary structure of DNA demonstrates chiral properties that can be identified by CD spectroscopy. Supplementary Figure S11-B illustrates the two distinctive peaks associated with ct-DNA, specifically a negative band at approximately 245 nm and a positive band at approximately 275 nm. The band at 245 nm is attributed to the inherent chirality in the helical structure, while the 275 nm band arises from the non-centrosymmetric stacking of nucleobase pairs.[14] Upon the addition of H-NS to ct-DNA, both bands exhibited an increase in their CD values. This observation suggests that the DBD influences the helical conformation

of DNA as well as the stacking arrangement of bases in the secondary structure.

References

(1) Sehnal, D.; Bittrich, S.; Deshpande, M.; Svobodová, R.; Berka, K.; Bazgier, V.; Velankar, S.; Burley, S. K.; Koča, J.; Rose, A. S. Mol* Viewer: modern web app for 3D visualization and analysis of large biomolecular structures. *Nucleic acids research* **2021**, *49*, W431–W437.

(2) Černý, J.; Božíková, P.; Svoboda, J.; Schneider, B. A unified dinucleotide alphabet describing both RNA and DNA structures. *Nucleic Acids Research* **2020**, *48*, 6367–6381.

(3) Altona, C. t.; Sundaralingam, M. Conformational analysis of the sugar ring in nucleosides and nucleotides. New description using the concept of pseudorotation. *Journal of the American Chemical Society* **1972**, *94*, 8205–8212.

(4) Riccardi, E.; Van Mastbergen, E. C.; Navarre, W. W.; Vreede, J. Predicting the mechanism and rate of H-NS binding to AT-rich DNA. *PLoS Comput. Biol.* **2019**, *15*, e1006845.

(5) Verhoeven, J. Glossary of terms used in photochemistry (IUPAC Recommendations 1996). *Pure and Applied Chemistry* **1996**, *68*, 2223–2286.

(6) Stefan, M. I.; Le Novère, N. Cooperative binding. *PLoS computational biology* **2013**, *9*, e1003106.

(7) Hill, A. V. The possible effects of the aggregation of the molecules of hemoglobin on its dissociation curves. *j. physiol.* **1910**, *40*, iv–vii.

(8) Gesztelyi, R.; Zsuga, J.; Kemeny-Beke, A.; Varga, B.; Juhasz, B.; Tosaki, A. The Hill equation and the origin of quantitative pharmacology. *Archive for history of exact sciences* **2012**, *66*, 427–438.

(9) Virtanen, P.; Gommers, R.; Oliphant, T. E.; Haberland, M.; Reddy, T.; Cournapeau, D.; Burovski, E.; Peterson, P.; Weckesser, W.; Bright, J.; others SciPy 1.0: fundamental algorithms for scientific computing in Python. *Nature methods* **2020**, *17*, 261–272.

(10) Strahs, D.; Schlick, T. A-tract bending: insights into experimental structures by computational models. *Journal of molecular biology* **2000**, *301*, 643–663.

(11) Sahoo, B. K.; Ghosh, K. S.; Bera, R.; Dasgupta, S. Studies on the interaction of diacetylcurcumin with calf thymus-DNA. *Chemical Physics* **2008**, *351*, 163–169.

(12) Ling, X.; Zhong, W.; Huang, Q.; Ni, K. Spectroscopic studies on the interaction of pazufloxacin with calf thymus DNA. *Journal of Photochemistry and Photobiology B: Biology* **2008**, *93*, 172–176.

(13) Barcellona, M.; Gratton, E. The fluorescence properties of a DNA probe: 4-6-Diamidino-2-phenylindole (DAPI). *European Biophysics Journal* **1990**, *17*, 315–323.

(14) Karidi, K.; Garoufis, A.; Tsipis, A.; Hadjiliadis, N.; den Dulk, H.; Reedijk, J. Synthesis, characterization, in vitro antitumor activity, DNA-binding properties and electronic structure (DFT) of the new complex cis-(Cl, Cl)[Ru II Cl 2 (NO+)(terpy)] Cl. *Dalton Transactions* **2005**, 1176–1187.
